# Minimum acceptable diet and associated factors among children aged 6–23 months during fasting days of orthodox Christian mothers in Gondar city, North West Ethiopia

**DOI:** 10.1186/s40795-022-00558-z

**Published:** 2022-08-10

**Authors:** Haregewoin Birhanu, Kedir Abdela Gonete, Melkamu Tamir Hunegnaw, Fantu Mamo Aragaw

**Affiliations:** 1 Ministry of Health, Nutrition officer, Central Gondar, Gondar, Ethiopia; 2 Department of Human Nutrition, Institute of Public Health, College of Medicine and Health Sciences, University of Gondar, Gondar, Ethiopia; 3 Department of Epidemiology and Biostatistics, Institute of Public Health, College of Medicine and Health Sciences, University of Gondar, Gondar, Ethiopia

**Keywords:** Minimum acceptable diet, Children, Fasting, Orthodox Christian, Ethiopia

## Abstract

**Background:**

Nutritional improvement through appropriate feeding practices is critical for young children's healthy growth and development. Even if children are exempted from fasting, their diets are influenced by the widespread fasting practices of their Orthodox Christian mothers. However, scientific evidence on a minimum acceptable diet (MAD) among children aged 6–23 months during the fasting period was limited. Therefore, this study aimed to assess the minimum acceptable diet and associated factors among children aged 6–23 months during the fasting period among Orthodox Christian mothers in Gondar city, Northwest Ethiopia.

**Method:**

A community-based cross-sectional study was conducted during the fasting season (Lent) from March 8, 20,121 to April 8, 2021. A total of 738 Orthodox Christian mothers with their children were selected by multistage sampling. A structured questionnaire was used to collect data among mothers to assess children’s MAD status. The degree of association between independent and dependent variables was assessed by using an odds ratio with a 95% confidence interval. Those variables with a *p*-value of less than 0.05 in the multivariable analysis were considered as a significant factor for MAD among children aged 6–23 months. Data were presented using texts, tables and figures.

**Results:**

The overall prevalence of MAD among children aged 6–23 months was 19.4% (95% CI: 16.40%-22.20). Having household wealth index of rich and middle (AOR = 4.39, 95% CI: 2.26,8.50) and (AOR = 3.25, 95% CI: 1.69,6.22), respectively, children aged from 12–17 months (AOR = 2.66, 95% CI: 1.43,4.92) and 18–23 months (AOR = 5.39, 95% CI: 2.93,9.95) respectively, Children who lived with a family member who consumed any time without keeping the fasting time(AOR = 1.79, 95% CI: 1.13,2.83) and mothers of young children who were married (AOR = 4.13, 95% CI: 1.29,13.23) have significant association with MAD.

**Conclusion:**

The practice of minimum acceptable diet was inadequate. Age of child, wealth status, marital status, and presence of family member who fed without keeping fasting time were significantly associated factors for MAD among children aged 6–23 months. Advocacy for appropriate feeding practice and meeting the MAD for children aged 6–23 months during the fasting period should also be strengthened targeting the unmarried women and those with poor households and giving awareness for mothers in collaboration with the respective religious leaders is highly recommended.

**Supplementary Information:**

The online version contains supplementary material available at 10.1186/s40795-022-00558-z.

## Introduction

Children under the age of two suffer from poor health and growth due to insufficient quantity and quality of supplemental foods and poor child feeding practices [1]. Suboptimal infant and feeding practices is highly common in low and middle-income countries resulting in a significant increase in mortality and morbidity [2, 3]. Suboptimal feeding practice is the leading cause of childhood malnutrition in developing countries which results in poor physical and mental development and childhood illnesses and death especially in infants and young children aged between 6 and 23 months [4–7].

Appropriate feeding practice is vital for young children aged between 6 and 23 months as it is a critical window for optimal growth and development of the child [8]. Children aged between 6 and 23 months are also at a higher risk of malnutrition, as breast milk alone is insufficient to provide all nutritional requirements, requiring the initiation of complementary feeding [7, 9]. In children under the age of two, nutritional diversity and meal frequency behaviors are determinants of health and growth as they are at risk for malnutrition, disease, and death [10].

Minimum acceptable diet is one of the most important indicators for evaluating infant and young child feeding (IYCF) practice that combines standards of dietary diversity and feeding frequency by feeding status [11]. Minimum acceptable diet is a useful proxy for inadequate nutrient intake, but is not valid for assessing nutrient intake or risk for overweight and obesity [12].

Meeting minimum dietary diversity (MDD) and minimum meal frequency (MMF) criteria have been difficult in many low-income countries, including Ethiopia, especially in the areas where there is food insecurity [13]. Global reports indicate that less than one-fourth of children aged 6–23 months receive the minimum acceptable diet for children aged 6 to 23 months was 8.6% in Dembecha [4], 6.1% according to Ethiopian demographic and health survey( EDHS) report [14], 8.8% in two agro-ecological zones of rural Ethiopia [15], 21.1% in Wolaita Sodo [16], and 8.4% in Gorche District [14, 17]. The magnitude of minimum dietary diversity (MDD, minimum meal frequency (MMF), and minimum acceptable diet in Southern Ethiopia were 3.8, 49.8, and 3.1%, respectively [18].

World health organization (WHO) recommends breastfed children aged 6–23 months should eat animal-source diets and vitamin A-rich fruits and vegetables on a daily basis. As a result, the minimum acceptable diet for breastfeed infants comprises four food groups (grain or tuber-based staple, animal-source food, vitamin A-rich fruit and vegetable) [11]. Children who are not breastfed should be fed four or five times a day, with one or two snacks as desired. Meal frequency is used to estimate the amount of energy consumed from foods other than breast milk. As a result, feeding frequency indicators for non-breastfed children comprise both milk feeds and solid or semi-solid feeds [11, 19].

In Ethiopia where Orthodox Christians make up about half of the population [20], Ethiopian Orthodox Christians practice extensive fasting, with common people fasting for at least 110–115 days per year and priests and other church officials fasting for a total of 220 days [21]. On fasting days in the Ethiopian Orthodox Tewahedo Church (EOTC), all food and drink are abstained from until noon or late. Even though, animal source foods (ASFs) supply high-quality protein, energy, and a variety of micronutrients to young children and play an important role in their growth, cognitive development, and health [22], ASFs, including milk and eggs, are avoided entirely during fasting times in the EOTC [23].

Although children exempted from following the fasting rule, their diets are influenced by the widespread fasting practices [24], some mothers do not prepare their food from animal sources as it could contaminate utensils for cooking family foods [25]. Studies also reported that mothers/caregivers’ fasting practice as one of the contributing factors not to attain the recommended child dietary diversity [24–26]. Due to fear of utensil contamination during family food preparation, mothers/caregivers who did not feed their children a diet containing animal products during fasting season were less likely to feed the required dietary diversity [26].

Nutritional improvement through appropriate complementary feeding practices is critical for young children's healthy growth, development [26–28]. This study was mainly focus on Orthodox Christian mothers, since their practice of fasting affects the nutritional status of their children. Evidence on a minimally acceptable diet and associated factors in children aged 6–23 months, particularly during fasting times, is limited. As a result, the purpose of this study was to determine the prevalence of a minimum acceptable diet and its associated factors among children aged 6–23 months in Gondar, Ethiopia, during the fasting seasons.

## Methods

### Study design, and setting

A community-based cross-sectional study was conducted during the fasting period from March 8, 2021, to April 8, 2021. This study was conducted in Gondar City, northwest Ethiopia. Gondar City is found 180 Kilometers from Bahirdar, and 748 km from Addis Ababa. It had a population of 351, 675 divided into 10 sub-cities and 24 kebeles (smallest administrative units in Ethiopia) [29]. The majority of the inhabitants practiced Ethiopian Orthodox Christianity, with 90.2% reporting that as their religion, while 8% of the population said they were Muslim and 1.1% were Protestant.

### Study population, sample size and sampling procedures

Infants and young children aged 6–23 months with mothers / caregivers who were orthodox religion followers and available during data collection period were included.

The sample size was calculated using the single population proportion formula by taking the level of confidence at 95%, margin of error of 3%, design effect of 2, non-response rate of 10%, and expected prevalence of 8.6% from study conducted to assess minimum acceptable diet practice in 2019 at Dembecha, North West Ethiopia [4]. Also we calculated a sample size for factors by, education, maternal knowledge, and consumption of ASF was considered by using double population proportion formula. Finally by comparing the sample size for prevalence and factors, the larger sample size (738) that is the first the prevalence’s sample was taken.

Regarding the sampling procedures, a multistage sampling technique was employed and a total of seven kebeles were selected from a total of 24 kebeles using simple random sampling technique. From the total selected kebeles total sample size was allocated proportionally from each kebeles. Systematic random sampling was used to select children with their mothers in the study area. The registered infants and young children aged between 6 and 23 months with their mothers at the health center were used as a sampling frame. Whenever more than one eligible respondent was found in the same selected household, only one respondent was chosen using the lottery method.

## Variables

### Dependent variables

Minimum Acceptable diet (Adequate / in adequate).

### Independent variables

#### Socio-demographic and economic factors

Maternal age, marital status, ethnicity, number of Children, family size, parental education, parental occupation, wealth index, HH food security.

#### Child Characteristics

Age, sex, birth order, child illness, breastfeeding.

#### Utilization of health care services

ANC, place of delivery, PNC, immunization.

#### Nutrition information

Being exposed to Medias like TV, Radio.

### Operational definitions

#### Complimentary food (CF)

Any solid, semi-solid, or soft food, whether manufactured or locally prepared, suitable as a complement to breast milk or infant formula, when either becomes insufficient to satisfy the nutritional requirements of the infant [30].

#### Minimum dietary diversity (MDD)

Those children 6–23 months of age who receive foods from four or more food groups during the previous day considered to have adequate MDD and if they receive less than four or more food groups during the previous day are considered as having inadequate MDD. The seven food groups used for tabulation of this indicator were: grains, roots, and tubers; legumes and nuts; dairy products (milk, yogurt, and cheese); flesh foods (meat, fish, poultry, and liver/organ meats); eggs; vitamin A-rich fruits and vegetables; and other fruits and vegetables [31].

#### Minimum meal frequency (MMF)

Those children both breastfed and non-breastfed children 6–23 months of age who receive solid, semi-solid, or soft foods the minimum number of times or more (minimum is defined as two times for breastfed infants 6–8 months; three times for breastfed children 9–23 months; and four times for non-breastfed children 6–23 months) in the previous day was considered as having adequate MMF, otherwise they will be considered as having inadequate MMF [4].

#### Minimum acceptable diet (MAD)

MAD is considered as adequate if children’s received the minimum acceptable diet (both minimum dietary diversity and minimum meal frequency) during the previous 24 h, otherwise it is considered as inadequate [4].

HFIAS (household food insecurity access scale):- was assessed from FANTA (Food and Nutrition Technical Assistance) 2007 with nine main question, HFIAS divided into food secure if the summations were ≤ 1 point out of 27 scores while the household food security level of the summations ≥ 2 points out of 27 scores were considered as food insecure [32].

#### Wealth index

It is a composite measure of a household's cumulative living standard. Generated with a statistical procedure known as principal components analysis, the wealth index places individual households on a continuous scale of relative wealth tertile (rich, middle, and poor) [33]*.*

#### Nutrition information

Those who have exposed to medias like TV, Radio was considered as having nutrition information while others who have not exposed to media were considered as not having nutrition information.

### Data collection tools, procedures, and quality control

The primary data on the practice of Minimum acceptable diet and associated factors was collected from mothers with a child aged 6–23 months and followers of the Orthodox Christian religion. The data collection tools were adopted from the FANTA tool and different kinds of literature. Five public health officers have participated as data collectors and two public health officers were participate as supervisors. An interviewer-administered questionnaire was used to collect data. The questionnaire was prepared in English and translated to Amharic by the local language expert and retranslated to English. A 5% pre-tested structured and standardized questionnaire was used to assure data quality. Training was given for the data collectors and supervisors for two days. The completeness of the questionnaire was checked before data entry.

### Data processing and analysis

The data was entered into Epi info version 7.0 and exported to SPSS version 20 for further data processing and analysis. To demonstrate the relationship between dependent and independent variables, a binary logistic and a multivariable logistics regression analysis was performed. Variables that had a *p*-value less than 0.2 in bivariable logistic regression were entered into a multivariable logistic regression to determine the effect of independent variables on dependent variables. Odds ratio with a 95% confidence interval to ascertain the association between independent and dependent variables was used. Finally, a *p*-value of less than 0.05 was used to declare Stastical significance [34].

### Ethical consideration

Ethical clearance was first obtained from the institutional review board of Institutes of public health, college of medicine and health sciences, University of Gondar. Written permission was taken from Gondar administrative office. Before the data collection begins, written informed consent was taken from each study participant (mothers/caregivers). The data collectors were informed each study participant about the purpose and anticipated benefits of the research project and the study participants were informed of their full right to refuse or completely reject part or all of their part in the study. All methods were carried out in accordance with relevant guidelines and regulations.

## Results

### Socio-demographic and economic characteristics of the study population

A total of 738 infants and young children aged between 6 and 23 months with their mothers were enrolled in the study. More than half (52.7%) of children were children were females and more than one-third (36.9%) were in the age between 18 and 23 months. The mean age of children was 14.8 ± 5.2 months. Majority (91.7%) of women were married. Two hundred and sixty-seven (36.3%) of fathers were government employed. Six hundred three (81.7%) of respondents were from food secured households, and about one-third (32.0%) of respondents were from low wealth index households (Table 1).

**Table 1:** Socio demographic and economic characteristics of parents and their children aged 6–23 months, Gondar City, North west Ethiopia, 2021(*n* = 738)

Variables	Mean with SD	Frequency	Percentage
Mother age( years)	29 ± 10		
**18–26**		205	27.8
**27–31**		342	46.3
**32–43**		191	25.9
Marital status			
**Married**		677	91.7
**Single**		28	3.8
**Separated**		15	2.0
**Divorced**		13	1.8
**Widowed**		5	0 .7
Mother Educational status			
**Unable to read and write**		68	9.2
**Able to read and write**		52	7
**Primary school (1—8)**		185	25.1
**Secondary school (9 – 12)**		265	35.9
**Collage and above**		304	41.2
Mother occupation			
**House wife**		497	67.3
**Government employed**		95	12.9
**Private employee**		30	4.1
**Merchant**		60	8.1
**Daily laborer**		56	7.6
Father occupation			
**Government employed**		267	36.2
**Private employee**		194	26.3
**Merchant**		137	18.6
**Daily laborer**		78	10.6
Family size			
**Less than three**		265	35.9
**Four to nine**		473	64.1
Decision making			
**Mothers not involved**		507	68.7
**Mothers involved**		231	31.3
House hold food security			
**Insecure**		135	18.3
**Secure**		603	81.7
Wealth index			
**Poor**		240	32.5
**Middle**		246	33.3
**Rich**		252	34.1
Age of the child( in months)	14.8 ± 5.2		
**6–11**		242	32.8
**12–17**		224	30.4
**18–23**		272	36.9
Sex of the child			
**Male**		389	52.7
**Female**		349	47.3
Child living status			
**Living with only their mother**		61	8.26
**Living with both mother and father**		677	91.73

### Health care service related characteristics

During pregnancy, around 649 (87.9%) mothers received ante-natal care service four,and above times. Seven hundred and thirteen (96.6%) mothers were delivered in health facility. While, more than half of mothers (54.6%) did not receive post natal care. Six hundred fifty one (88.2%) of children were breastfed (Table 2).

**Table 2:** Health care service characteristics among children aged 6–23 months and their mothers at Gondar City, North Wet Ethiopia, 2021 (*n* = 738)

Characteristics	Frequency	Percentage
Number of ANC		
No ANC	13	1.8
One to three	76	10.3
Four and above four	649	87.9
**Birth place**		
Government health facility	713	96.6
Home delivery	22	3
Private health facility	3	0.4
**Birth order**		
First	299	40.5
Second to third	352	47.7
Fourth to fifth	77	10.4
Above sixth	10	1.4
**Birth Interval (in months)**		
12–35	133	18.0
36- 59	141	19.1
60 and above	167	22. 6
**vaccination status**		
Not started	3	0.4
Defaulter	25	3.4
Started	348	47.2
Completed	362	49.1
**Currently breast feed**		
Yes	651	88.2
No	87	11.8
**Postnatal care service**		
Yes	335	45.4
No	403	54.6
**Time of CF initiation**		
Before 6 month	206	27.9
At 6 month	482	65.3
After 6 month	50	6.8
**Current illness**		
RTI	120	16.3
Fever	36	4.9
Diarrhea	33	4.5
Abdominal pain	16	2.2
Vomiting	13	1.8
Other	20	2.7

*ANC* Antenatal care, *CF* Complementary feeding, *RTI* Respiratory tract infection

### Nutrition and fasting related characteristics

About one third (35.6%) of mothers of children had got information on young child dietary practice. Seven hundred eight (95.9%) of children lived in a family who were not consumed animal source foods. Two third (59.6%) of children lived in a family who consumed food at any time without keeping the fasting time (Table 3).

**Table 3:** Nutrition information and fasting related characteristics of families of children aged 6–23 months at Gondar City, North West Ethiopia, 2021(*n* = 738)

Characteristics	Frequency	Percentage
**Read newspapers and magazine**		
No	569	77.1
One and above times a week	169	22.9
**Information on dietary diversity and meal frequency**		
Yes	263	35.6
No	475	64.4
**Information about when to start complementary feeding**		
Yes	722	97.8
No	1 6	2.2
**Information on child feeding**		
Yes	264	35.8
No	474	64.2
**Animal source foods**		
Consume animal source foods	30	4.1
Not consume animal source foods	708	95.9
**keeping fasting time**		
Consume at any time in fasting	440	59.6
Not consume at any time in fasting	298	40.4

### Practice of minimum acceptable diet

**Fig. 1:**
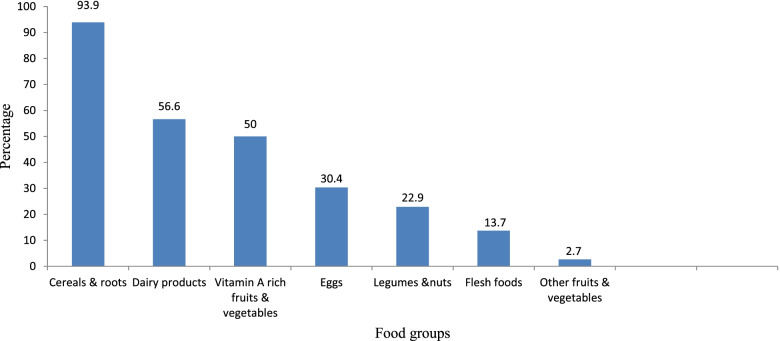
Food Groups Consumed by children aged 6–23 months at Gondar City, North West Ethiopia, 2021(*n* = 738)

**Fig. 2:**
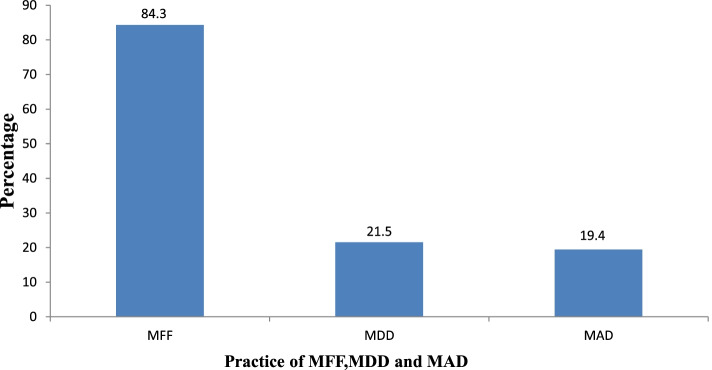
Practice of minimum acceptable diet, minimum dietary diversity and minimum meal frequency among children aged between 6–23 months at Gondar City, North West Ethiopia, 2021(*n* = 738)

From all children, Six hundred ninety three (93.9%) of children received cereals and tubers followed by dairy products which were consumed by more than half of (56.6%) of the children (Fig. 1). Six hundred twenty two (84.3%) of children received minimum meal frequency. One hundred fifty nine (21.5%) of children received minimum dietary diversity and one hundred forty three (19.4%) with 95% CI (16.4%, 22.2) children had minimum acceptable diet (Fig. 2).

### Factors associated with minimum acceptable diet

From all variables entered to the regression model only maternal age, maternal education, maternal occupation, Father education, father occupation, marital status, nutrition information, wealth index, complementary feeding demonstration, time of complimentary food initiation, house hold food security, child sex, child age, birth order, current breast feeding status, postnatal care presence of family member who fed ASF and without keeping fasting time were significant for MAD during bivariable regression using *p* value less than 0.2.

In the multivariable logistic regression analysis, age of the child, household wealth status, marital status and presence of family members who fed without keeping fasting time were significantly associated with MAD of children aged 6–23 months.

Children with age from 12–17 months adjusted odd ratio (AOR) 2.66; 95% CI 1.43, 4.92 and 18–23 months AOR 5.39, 95% CI: 2.93, 9.95 respectively were more likely to receive the recommended MAD than younger children. Households with rich and middle wealth index were more likely to feed the recommended MAD to their children AOR 4.39, 95% CI: 2.26, 8.50 and AOR 3.25, 95% CI: 1.69, 6.22, respectively as compared to those with poor wealth index households.

Mothers who were married were more likely to give recommended MAD to their children (AOR = 4.13, 95% CI: 1.29, 13.23) than those who were single mothers. Children who lived with a family member who consumed any time without keeping the fasting time were more likely to receive a minimum acceptable diet (AOR = 1.79, 95% CI: 1.13, 2.83), than those children who were living in houses with no family member consumed any time without keeping the fasting time (Table 4).

**Table 4:** Bivariable and multivariable regression results of MAD for children aged 6–23 months at Gondar City, Northwest Ethiopia, 2021(*n* = 738)

Variables	MAD status			
	**Adequate**	**Inadequate**	**COR (95% CI)**	**AOR (95% CI)**
Age of mother in years				
18–26	14(15.4%	77(84.6%	1	1
27–31	90(18.0%	409(82.0%	1.21(0.65–2.23)	1.17(0.57–2.43)
32–43	39(26.4%	109(73.6%	1.97 (1.00–3.87)	1.79(0.72–4.50)
Marital status				
Married	138(2.4%	539(79.6%	2.87 (1.23–7.29)	4.13(1.29–13.23)
Unmarried	5(8.2%	56(91.8%	1	1
Mother Educational status				
Unable to read and write	11(16.2%	57(83.8%	1	1
read and write	15(28.8%	37(71.2%	2.10 (0.87–5.07)	2.07(0.72–5.98)
Primary (1—8)	27(14.6%	158(85.4%	0.89 (0.41–1.90)	1.06(0.42–2.68)
Secondary (9 -12)	42(15.8%	223(84.2%	0.98(0.47–2.01)	1.25(0.49–3.12)
Certificate and above	48(28.6%	120(71.4%	2.07(1.00–4.29)	2.63(0.96–7.16)
Mother occupation				
House wife	90(18.1%	407(81.9%	1.68(0.84–3.38)	1.24 (0.55–2.80)
Government	26(27.4%	69(72.6%	2.86(1.29–6.37)	0.77(0.29–2.05)
Merchant	17(28.3%	43(71.7%	3.00(1.26–7.14)	2.42(0.86–6.76)
Private	10(11.6%	76(88.4%	1	1
Wealth index				
Poor	17(7.1%	223(92.9%	1	1
Middle	47(19.1%	199(80.9%	3.09(1.72–5.57)	3.25 (1.69–6.22)
Rich	79(31.3%	173(68.7%	5.99(3.42–10.49))	4.39 (2.26–8.50)
HFSA				
Insecure	18(13.3%	117(86.7%	1	1
Secure	125(20.7%	478(79.3%	1.70(0.99–2.89)	1.50(0.78–2.89)
Age of the child in months				
6–11	24(9.9%	218(90.1%	1	1
12–17	41(18.3%	183(81.7%	2.03(1.18–3.49)	2.66(1.43–4.92)
18–23	78(28.7%	194(71.3%	3. 65 (2.22–6.00)	5.39(2.93–9.95)
Sex of the child				
Female	75(21.5%	274(78.5%	1	1
Male	68(17.5%	321(82.5%	0.77(0.54–1.11)	0.75 (0.49–1.15)
Birth order				
First	56(18.7%	243(81.3%	1	1
Second	38(16.0%	200(84.0%	0.82(0.52–1.29)	0.87(0.50–1.49)
Third and Above	49(24.4%	152(75.6%	1.39(0.91–2.16)	0.86(0.46–1.60)
Currently breast feed				
No	30(34.5%	57(65.5%	1	1
Yes	113(17.4%	538(82.6%	0.39(0.24–0.65)	0.81(0.44–1.51)
Postnatal care				
No	56(13.9%	347(86.1%	1	1
Yes	87(26.0%	248(74.0%	2.17(1.49–3.16)	1.52(0.95–2.43)
Time of CF initiation				
Before 6 month	50(24.3%	156(75.7%	1	1
At 6 month	86(17.8%	396(82.2%	0.68(0.46–1.01)	0.75(0.46–1.20)
After 6 month	7(14.0%	43(86.0%	0.51(0.21–1.20	0.98(0.36–2.67)
Nutrition information				
**No**	64(13.5%	414(86.5%	1	1
**Yes**	79(30.0%	184(70.0%	2.76(1.89–4.00)	1.68 (1.01–2.81)
complementary feeding demonstration				
**No**	93(15.0%	527(85.0%	1	1
**Yes**	50(42.4%	68(57.6%	4.18(2.72–6.38)	1.98(1.05–3.74)
Other than child ASF consumption				
**No**	130(18.4%	578(81.6%	1	1
**Yes**	13(43.3%	17(56.7%	3.40(1.61–7.17)	1.99(0.77–5.15)
Other than child any time consumption				
**No**	42(14.1%	256(85.9%	1	1
**Yes**	101(23.0%	339(77.0%	1.82(1.22–2.69)	1.79 (1.13–2.83)

^*^Statistically significant at *p*-value < .0.05

*AOR* Adjusted odd ratio, *ASF* Animal source food, *OR* Crude odd ratio, *HFSA* Household food security access, *MAD* Minimum acceptable diet

## Discussion

This study assessed MAD and associated factors among children aged 6–23 months, whose mothers were Orthodox Christians during the fasting season. In this study, about 19.4% of the children surveyed feed MAD. This finding was in line with the study conducted in Myanmar (16.00%) [31] and Delhi (19.70%) [35]. However, it is higher than studies conducted in different parts of Ethiopia such as Tigray (2.30%) [25], EDHS 2016( 6.10%) [14], Dembecha (8.60%) [4] and, East Gojam zone Goncha district (8.40%) [36]. On the other hand the result of this study is lower than other studies from central Amhara (31.60%) [37], Kaski (42.40%) [38], Abu Dhabi(36.20%) [39], Ghana (24.90%) [40] and Bangladesh (23.00%) [41]. The possible reasons for the variation might be due to differences in a study setting, different socioeconomic statuses, and seasonal difference in data collection. Furthermore, this study was conducted among orthodox religion followers during the fasting season, when dietary patterns may be reduced in terms of food diversity and meal frequency, underestimating the findings when compared to other times.

Those children having a married mother was positively associated with recommended minimum acceptable diet similar to another study done in Tigray, Ethiopia [42]. The possible reason might be marriage has the important role in sustaining better life and economic wellness which will contribute to the better wellbeing of children including a minimum acceptable diet.

This study found that children in the 12–17 and 18–23 month age groups were more likely to receive the recommended number and variety of feeds, compared to children aged between 6 to 11 months this finding is similar with studies done in Ethiopia and India [10, 37, 43–46]. The justification for this might be as the child’s age increased the chance that they will exposed to a diversified diet will be increased.

Those mothers with the highest and middle wealth index were more likely to feed the recommended MAD to their children as compared to their low wealth index counterparts. This finding is consistent with study conducted in Ethiopia [26, 36, 44]. The possible explanation of this significance association might be due to the limited food purchasing power to provide diversified diet and adequate amount of food to their children in peoples with lower wealth index. Children from households with a high level of income ate a more diverse diet than those from households with a low level of income.

This study also showed that children who lived with a family member who consumed any time without keeping the fasting time were more likely to receive a MAD than those children who were living in houses with no family member who consumed any time without keeping the fasting time. This might be due to mothers become reluctant to prepare a separate meal as early as possible, even some times mothers may late to prepare food for their young child up to the mid-day if there is no another person in the house who eat in the morning.

Other important variables like ANC, HFSA, and family size were not significantly associated with having adequate MAD. The possible reason might be that these factors may not have an effect on the child’s opportunity to receive MAD as it was mainly on the orthodox Christian mothers and the factors may be mainly related to the fasting practice.

Our study is limited to show the differences by comparing dietary practice of both fasting and non-fasting participants during fasting and non-fasting periods and the study used only 24-h recall method which tells us only one time phenomenon but did not demonstrate dietary habit of the participants. Also the study may not free from social desirability bias in responding to questions on the type and frequency of foods given to children.

## Conclusion

The practice of minimum acceptable diet was inadequate. Age of child, wealth status, marital status, and presence of family member who fed without keeping fasting time were significantly associated factors for MAD among children aged 6–23 months. Advocacy for appropriate feeding practice and meeting the MAD for children aged 6–23 months during the fasting period should also be strengthened targeting the unmarried women and those with poor households and giving awareness for mothers in collaboration with the respective religious leaders is highly recommended.

## Supplementary Information

**Additional file 1.**

## Data Availability

The data that support the findings of this study are available from the corresponding author but restrictions apply to the availability of these data, which were used under license for the current study, and so are not publicly available. Data are however available from the authors upon reasonable request and with permission of the corresponding author. The English version of the data collection tool was available on supplementary file.

## Abbreviations

ANC: Antenatal Care
ASF: Animal Source Food
CF: Complementary Feeding
CSA: Central Statistical Agency
DDS: Dietary Diversity
DHS: Demographic and Health Survey
EDHS: Ethiopian Demographic and Health Survey
HEW: Health Extension Workers
IYCF: Infant and Young Child Feeding
MDD: Minimum Dietary Diversity
MMF: Minimum Meal Frequency
MAD: Minimum Acceptable Diet
MOH: Ministry Of Health
PNC: Post-Natal Care
SPSS: Statistical Package for Social Science
UNICEF: United Nation Children’s Fund
WHO: World Health Organization

## References

[1] Black RE, Allen LH, Bhutta ZA, Caulfield LE, De Onis M, Ezzati M (2008). Maternal and child undernutrition: global and regional exposures and health consequences. Lancet.

[2] Onyango AW, Borghi E, de Onis M, del Carmen Casanovas M, Garza C (2014). Complementary feeding and attained linear growth among 6–23-month-old children. Public Health Nutr.

[3] Organization WH. 2009. Infant and young child feeding: model chapter for textbooks for medical students and allied health professionals: World Health Organization. 23905206

[4] Mulat E, Alem G, Woyraw W, Temesgen H. Uptake of minimum acceptable diet among children aged 6–23 months in orthodox religion followers during fasting season in rural area, DEMBECHA, north West Ethiopia. BMC Nutr. 2019;5(1):1–10. 10.1186/s40795-019-0274-y PMC7050747 32153931

[5] Tette EM, Sifah EK, Tete-Donkor P, Nuro-Ameyaw P, Nartey ET. Feeding practices and malnutrition at the Princess Marie Louise Children’s hospital, Accra: what has changed after 80 years? BMC Nutr. 2016;2(1):1–10.

[6] Rah JH, Akhter N, Semba RD, De Pee S, Bloem MW, Campbell AA (2010). Low dietary diversity is a predictor of child stunting in rural Bangladesh. Eur J Clin Nutr.

[7] Dewey K (2003). Guiding principles for complementary feeding of the breastfed child.

[8] Daelmans B, Ferguson E, Lutter CK, Singh N, Pachón H, Creed‐Kanashiro H, et al. Designing appropriate complementary feeding recommendations: tools for programmatic action. Matern Child Nutr. 2013;9:116-30. 10.1111/mcn.12083 PMC6860844 24074322

[9] Khanal V, Sauer K, Zhao YJ. Determinants of complementary feeding practices among Nepalese children aged 6–23 months: findings from demographic and health survey 2011. BMC Pediatr. 2013;13(1):1–13. 10.1186/1471-2431-13-131 PMC3766108 23981670

[10] Beyene M, Worku AG, Wassie MM. Dietary diversity, meal frequency and associated factors among infant and young children in Northwest Ethiopia: a cross-sectional study. BMC Public Health. 2015;15(1):1-9. 10.1186/s12889-015-2333-x PMC4592571 26433689

[11] Organization WH (2010). Indicators for assessing infant and young child feeding practices part 3: country profiles.

[12] Lele U, Masters WA, Kinabo J, Meenakshi J, Ramaswami B, Tagwireyi J, et al. Measuring food and nutrition security: An independent technical assessment and user’s guide for existing indicators. Food Secur Inform Netw. 2016;177.

[13] Beyene M, Worku A, Wassie M. Dietary diversity, meal frequency and in Northwest Ethiopia: A cross-sectional study associated factors among infant and young children. BMC Public Health. 2015;15:1007-16. 10.1186/s12889-015-2333-x PMC4592571 26433689

[14] Tassew AA, Tekle DY, Belachew AB, Adhena BM (2019). Factors affecting feeding 6–23 months age children according to minimum acceptable diet in Ethiopia: a multilevel analysis of the Ethiopian Demographic Health Survey. PLoS One.

[15] Roba KT, O’Connor TP, Belachew T, O’Brien NM. Infant and young child feeding (IYCF) practices among mothers of children aged 6–23 months in two agro-ecological zones of rural Ethiopia. Int J Nutr Food Sci. 2016;5(3):185–94.

[16] Mekonnen TC, Workie SB, Yimer TM, Mersha WF, Population, Nutrition (2017). Meal frequency and dietary diversity feeding practices among children 6–23 months of age in Wolaita Sodo town. Southern Ethiopia.

[17] Dangura D, Gebremedhin S. Dietary diversity and associated factors among children 6–23 months of age in Gorche district, Southern Ethiopia: Cross-sectional study. BMC Pediatr. 2017;17(1):1–7. 10.1186/s12887-016-0764-x PMC5223415 28068965

[18] Ababa A, Calverton EJE, Demographic CE, Survey H. Central statistical agency (Ethiopia) and ICF international. 2011.

[19] Organization WH (2008). Indicators for assessing infant and young child feeding practices: part 1: definitions: conclusions of a consensus meeting held 6–8 November 2007 in Washington DC.

[20] Commission PC. Summary and statistical report of the 2007 population and housing census. Population size by age and sex. Canada: Springer; 2008.

[21] Knutsson KE, Selinus R. Fasting in Ethiopia: An anthropological and nutritional study. Am J Clin Nutr. 1970;23(7):956-69. 10.1093/ajcn/23.7.956 5465986

[22] Hoppe C, Mølgaard C, Michaelsen KF (2006). Cow's milk and linear growth in industrialized and developing countries. Annu Rev Nutr.

[23] Bazzano AN, Potts KS, Mulugeta A. How do pregnant and lactating women, and young children, experience religious food restriction at the community level? A qualitative study of fasting traditions and feeding behaviors in four regions of Ethiopia. PLoS One. 2018;13(12):e0208408. 10.1371/journal.pone.0208408 PMC6281271 30517203

[24] Kim SS, Nguyen PH, Tran LM, Abebe Y, Asrat Y, Tharaney M (2019). Maternal behavioural determinants and livestock ownership are associated with animal source food consumption among young children during fasting in rural Ethiopia. Matern Child Nutr.

[25] Desalegn BB, Lambert C, Riedel S, Negese T, Biesalski HK (2019). Feeding practices and undernutrition in 6–23-month-old children of Orthodox Christian mothers in rural Tigray, Ethiopia: longitudinal study. Nutrients.

[26] Kumera G, Tsedal E, Ayana M. Dietary diversity and associated factors among children of Orthodox Christian mothers/caregivers during the fasting season in Dejen District, North West Ethiopia. Nutr Metab. 2018;15(1):1-9. 10.1186/s12986-018-0248-0 PMC5813384 29456587

[27] Organization WH. Strengthening action to improve feeding of infants and young children 6-23 months of age in nutrition and child health programmes: report of proceedings, Geneva, 6-9 October 2008. 2008.

[28] Marriott BP, White A, Hadden L, Davies JC, Wallingford JC. World Health Organization (WHO) infant and young child feeding indicators: associations with growth measures in 14 low‐income countries. Matern Child Nutr. 2012;8(3):354–70. 10.1111/j.1740-8709.2011.00380.x PMC6860880 22171937

[29] Finance G. Economy directory (2017) Population Census projection in 2017. Gondar Finance and Economic office.

[30] United Nations Children's Fund (UNICEF). Improving young children's diets during the complementary feeding period. UNICEF programming guidance. 2020

[31] Mya KS, Kyaw AT, Tun T (2019). Feeding practices and nutritional status of children age 6–23 months in Myanmar: a secondary analysis of the 2015–16 demographic and health survey. PLoS One.

[32] Worku T, Gonete KA, Muhammad EA, Atnafu A. Sustainable under nutrition reduction program and dietary diversity among children’s aged 6–23 months, Northwest Ethiopia: Comparative cross-sectional study. Int J Equity Health. 2020;19(1):1–11. 10.1186/s12939-019-1120-1 PMC6986108 31992299

[33] Gonete KA, Tariku A, Wami SD, Derso T. Prevalence and associated factors of anemia among adolescent girls attending high schools in Dembia District, Northwest Ethiopia, 2017. Arch Public Health. 2018;76(1):1–9. 10.1186/s13690-018-0324-y PMC6302287 30598822

[34] Guirindola MO, Maniego MLV, Silvestre CJ, Acuin CCS. Determinants of meeting the minimum acceptable diet among Filipino children aged 6–23 months. Philipp J Sci. 2018;147(March):75–89.

[35] Khan AM, Kayina P, Agrawal P, Gupta A, Kannan AT (2012). A study on infant and young child feeding practices among mothers attending an urban health center in East Delhi. Indian J Public Health.

[36] Birie B, Kassa A, Kebede E, Terefe B. Minimum acceptable diet practice and its associated factors among children aged 6–23 months in rural communities of Goncha district, north West Ethiopia. BMC Nutr. 2021;7(1):1–9. 10.1186/s40795-021-00444-0 PMC8290531 34281613

[37] Molla A, Egata G, Getacher L, Kebede B, Sayih A, Arega M (2021). Minimum acceptable diet and associated factors among infants and young children aged 6–23 months in Amhara region Central Ethiopia: community-based cross-sectional study. BMJ Open.

[38] Khatri D, Shrestha N. Factors Associated with Feeding Practices of Children in Kaski. J Health Allied Sci. 2016;5(1):14-20.

[39] Taha Z, Garemo M, Nanda J. Complementary feeding practices among infants and young children in Abu Dhabi, United Arab Emirates. BMC Public Health. 2020;20(1):1-8. 10.1186/s12889-020-09393-y PMC7453515 32854658

[40] Saaka M, Larbi A, Mutaru S, Hoeschle-Zeledon I. Magnitude and factors associated with appropriate complementary feeding among children 6–23 months in northern Ghana. BMC Nutr. 2016;2(1):1–8.

[41] Sheikh N, Akram R, Ali N, Haque SR, Tisha S, Mahumud RA (2020). Infant and young child feeding practice, dietary diversity, associated predictors, and child health outcomes in Bangladesh. J Child Health Care.

[42] Buzuayehu B. Minimum acceptable diet practice and its associated with household food security status (access) a, pmg cjo; drem 6–23, pmtj pf age om Tigray, Ethiopia: ACIPH; 2016.

[43] Berhe Gebremichael GE, Assefa N. Dietary diversity practice and associated factors among infants and young children in Haramaya town, Ethiopia. Int J Public Health Sci. 2017;6(3):243-50.

[44] Belew AK, Ali BM, Abebe Z, Dachew BA. Dietary diversity and meal frequency among infant and young children: a community based study. Ital J Pediatr. 2017;43(1):1-10. 10.1186/s13052-017-0384-6 PMC5558775 28810887

[45] Abebe H, Gashu M, Kebede A, Abata H, Yeshaneh A, Workye H, et al. Minimum acceptable diet and associated factors among children aged 6–23 months in Ethiopia. Ital J Pediatr. 2021;47(1):1–10. 10.1186/s13052-021-01169-3 PMC8557568 34717712

[46] Acharya A, Pradhan MR, Das AK. Determinants of Minimum Acceptable Diet Feeding among Children Aged 6–23 Months in Odisha, India. Public Health Nutr. 2021:1–27. 10.1017/S1368980021002172 PMC10195256 34034833

