# Supplementary material for: Minimum acceptable diet and associated factors among children aged 6–23 months during fasting days of orthodox Christian mothers in Gondar city, North West Ethiopia
Source: BMC Nutr. 2022 Aug 10;8:76. doi: 10.1186/s40795-022-00558-z (PMC9364522; doi:10.1186/s40795-022-00558-z)
Supplement: Supplementary file 1 — Additional file 1. [file 40795_2022_558_MOESM1_ESM.docx]

**English Questionnaire**

**A questionnaire prepared to assess the minimum acceptable diet practice of young children during fasting days among orthodox Christian mothers in Gondar town.**

INTRODUCTION AND CONSENT

The main aim of his study is to assess Young child minimum acceptable diet practice. The results of the study will be used as baseline information to design appropriate intervention strategies to improve the minimum acceptable diet practice of young children. Your name will not be written in this form and the information you give is kept confidential. If you do not want to answer all or some of the questions, you do have the right to do so. However, your willingness to answer all of the questions would be appreciated. If you have any questions, don’t hesitate to ask the interviewer. It doesn’t take more than 30 minutes.

Would you participate in responding to the questions in this questionnaire?

Yes --------No------

Questionnaire

Keble___________ Interview Number___________________

Date of Data Collection_____________________

Part I: Socio demographic characteristics

| SN. | Questions | Response | | | | | | | | | | |
| --- | --- | --- | --- | --- | --- | --- | --- | --- | --- | --- | --- | --- |
| 101 | Age of mother/caregiver | ____________________Years | | | | | | | | | | |
| 102 | Ethnicity | 1. Amhara | | 2.Tigre | | | | 3.Oromo | | | 4.Others_____ | |
| 103 | Marital status of mother | 1. Married | 2. Unmarried | | 3Separated | | | | 4.Divorced | | | 5.Widowed |
| 104 | Maternal Education | 1. Unable to read and write  2. Able to read and write | | | | 3. Primary (1 - 8)  4. Secondary (9 - 12) | | | | | | 5.Certificate and above |
| 105 | Father Education | 1. Unable to read and write  2. Able to read and write | | | | 3. Primary (1 - 8)  4. Secondary (9 - 12) | | | | | | 5.Certificate and above |
| 106 | Mother Occupation | 1.House wife  2.Government employed | | | | | 3.Private employee/NGO  4.Merchant | | | 5. Daily laborer  6.Others specify____ | | |
| 107 | Father Occupation | 1Government employed  2Private employee/NGO | | | | | 3Merchant  4Daily laborer | | | 5Others______ | | |
| 108 | Family Size | ____________________ | | | | | | | | | | |
| 109 | Child age(months) | ____________________ | | | | | | | | | | |
| 110 | Child Sex | 1Male 2. Female | | | | | | | | | | |

**Part II: Maternal and child health service related questions**

| **SN** | **Questions** | **Response** | | | | **Skip** | | |  |
| --- | --- | --- | --- | --- | --- | --- | --- | --- | --- |
| 201 | Birth Order | _________________________ | | | |  | | |  |
| 202 | Number of children |  | | | |  | | |  |
| 203. | If Q#202 is one child, skip Q#203  Birth interval for the last children? | _________________________ | | | |  | | |  |
| 204 | ANC for the last child | Yes | | 0. No | |  | | |  |
| 205. | The answer is “Yes” for Q#204, Number of ANC visit is? | __________________________ | | | |  | | |  |
| 206. | Place of birth for the last child | 1Home without Traditional birth attendant  2Home with Traditional birth attendant  3Government health facility  4Private health facility | | | |  | | |  |
| 207 | Postnatal visit for the last delivery? | 1. Yes | | 0. No | |  | | |  |
| 208. | The answer is “Yes” for Q#207; information/ advised about complementary feeding after delivery. | Yes | | 0. No | |  | | |  |
| 209. | The answer is “Yes” for Q#207; what information about child feeding during the postnatal visits.  (**More than one answer is possible**) | 1.When to start Complementary feeding  2.Dietary diversity  3.meal frequency  4.Breast feeding | | | | 1. Other specify | | |  |
| 210. | Growth monitoring and promotion service given for the current child monthly? | 1.Yes | 0. No | | |  | |  |  |
| 211 | Vaccination status | 1. Not started | 2.Partial | | | 3. Complete | | | |
| 212. | Vit. A supplementation in the past six Months | Yes | | 0. No | | |  | | |
| 213. | Child illness during the last two weeks | Yes | | 0. No | | |  | | |
| 214 | If yes Q#214, which type of illness | _____________________ | | | | |  | | |
| 215 | During illness, has the child feeding practice changed If “No” skip Q# 217 and Q#218. | 1.Yes | | 0. No | | |  | | |
| 216 | How could the practice changed? | 1. Preventing from breast  2. Preventing from giving food | | | 3. Providing additional food  4.Others______ | |  | | |
| 217 | How did you usually treat your child when get sick? | 1.Usually home treatment  2. Taking to traditional healers  3.Takingtohealth institution  4.Others______ | | |  | |  | | |
| 218 | Have you ever take your child to health Institution during sickness? | 1.Yes | | 0. No | | |  | | |

**Part III: Information about complimentary feeding**

| **SN** | **Questions** | **Response** | | | | | | | | | **Skip** |
| --- | --- | --- | --- | --- | --- | --- | --- | --- | --- | --- | --- |
| 301. | Ever breastfeeding | Yes 0. No | | | | | | | | |  |
|  | Current breastfeeding | Yes 0.No | | | | | | | | |  |
| 302. | Information about when to start Complementary feeding after birth | Yes 0.No | | | | | | | | |  |
| 303. | If “Yes” for Q#302, from where/whom do you get? ( **You can chose more than one** ) | From HEWs  Nurses/midwifery  From Television | | | | From Radio  Magazine  Other specify ______ | | | | |  |
| 304. | Do you have initiated complementary feeding? | 1. Yes 0. No | | | | | | | | |  |
| 305 | Number of feeding per day for the child | __________________? | | | | | | | | |  |
| 306. | If “Yes” for Q# 304, at what age of the child had complementary feeding began? | _______________months?  I don’t know | | | | | | | | |  |
| 307. | If “No” for Q# 304; what is the reasons?  (**More than one answer is possible**) | Age of the child is below six months  I don’t know when to initiate  Breast milk is enough for baby | | | | Child’s stomach can’t digest food  Not allowed with the community/culture  Specify any reason___ | | | | |  |
| 308. | If you initiate before 6 months of age,  What is the reason you initiate early? | I don’t know when to start  Breast milk is not enough for baby | | | | Mother return to work  No answer  Other(Specify)_____ | | | | |  |
| 309. | Did you give any food before six month | Yes 0. No | | | | | | | | |  |
| 310. | Which of the following food items did you initiate at first time in the last child?  ***(for those who began to feed other foods with or without breast milk)*** | Water  Tea  Cow’s milk | | Powdered milk  Cereal based fluid (such as porridge) | | | | 6.Adultfood(suchas Enjera,bread)  7.Other(Specify) | | |  |
| 311. | Child feeding style | 1,Bottle feeding | | | 2.Cup | | Spoon | | 4.Others | |  |
| 312. | Care of baby feeding | Mother/caregiver  Fathers | Sister  Grand mother | | | | 4.House maid | | | 5.Other specify_____ |  |

**Part IV: Dietary Assessment using 24hr recall and child characters’**

| Instruction: Ask the mother to recalls all the foods and beverages consumed yesterday during the day and night, whether at home or outside the home., underline the corresponding foods in the list under the appropriate food group” in the column next to the food group if at least one food in this group has been underlined. Once the recall is finished, probe for food groups where no food was underlined.  **Dietary Assessment using 24hr recall and child characters’** | | | |
| --- | --- | --- | --- |
|  | Food groups | Now I would like to ask you about foods your child ate yesterday during the day or night, either separately or combined with other foods. | |
|  |  |  | 1. Yes 0. No |
| 401 | Grain, root & tubers | Bread, Pasta, Rice, Noodles, Biscuits, Cookies, or any other food made from maize, barely, wheat, sorghum, millet or other grain? Or Any white potatoes, white yams, bulla, or any other foods made of roots? | Yes 0. No |
| 402 | Legumes& nuts | Any food made from lentils, beans, peas, ground nuts, peanuts, or sunflower seeds | Yes 0. No |
| 403 | Dairy product | Any milk cheese or yogurt? | Yes 0. No |
| 404 | Flesh foods | Any liver, kidney, heart, or other organ meat? Any beef, lamb, goat? Any chicken? Any fresh or dried fish? | Yes 0. No |
| 405 | Eggs | Egg or any food made with eggs | Yes 0. No |
| 406 | V A rich fruits & vegetables | Any pumpkin, carrots, or sweet potatoes that are yellow or orange inside? Any dark green leafy vegetable like kale, spinach? Any ripe mangoes, papayas, guavas? | Yes 0. No |
| 407 | fruits& vegetables | Any other fruit of vegetable? | Yes 0. No |

**Part V. Information about different factors of MAD**

| **SN** | **Questions** | **Response** | | | | | |
| --- | --- | --- | --- | --- | --- | --- | --- |
| 501 | Have you read nutrition related magazine, newspaper? | 1. Yes 0. No | | | | | |
| 502 | If you say “Yes” Q#501; how many times you read per a week? | ________________________? | | | | | |
| 503 | Do you know that the child should take diversified diet at any time? | 1. Yes 0. No | | | | | |
| 504 | Do you believe that the child should not take animal products during fasting season? | Yes 0. No | | | | | |
| 505 | If you say Yes for Q#504, why? | The food item is not necessarily for the child at this age.  The food item may mix with other foods.  The child should not take it because it is fasting season and fear of GOD. | | | | | |
| 506 | If you are recommended to give animal products to the child at this time by the following people at different time and place, whose recommendation will be implemented? | health extension workers  health worker from health centers  religion leaders  husband/ wife  kebele administrator  Other specify… | | | | | |
| 507 | If you are given an advice to feed the child with appropriate frequency based on the age by the following people whose advice is selected? | Health extension workers  Health worker from health centers  Religion leaders  Husband/wife  kebele administrator  Other specify__________ | | | | | |
| 508 | Is there discussion on Infant and young child feeding practice on female health development army meeting? | 1.Yes | 0.No | | | | |
| 509 | If “Yes”for Q#508; what agreement was shared by the health development army on feeding infants and young child with animal products and meal frequency during fating and non-fasting season? | Always similar feeding practice  Not give animal product during fasting  Decrease feeding frequency during fasting | | | | | |
| 510 | Is there any education or information you get on the Importance of feed the child with diversified food and feeding frequency? | 1. Yes | 0.No | | | | |
| 511 | If “Yes” for Q# 510 by what means you gets Information and education? | 1television/radio  2friend  3family member  4health professionals | | 5relative  religion leaders  6Other specify_______ | | | |
| 512 | Did you get practical observation of training on food preparation with different food items? | 1.Yes | 0.No | | | | |
| 513 | If “Yes” for Q#512, where you get? | 1.Television  2.health professionals | | | |  |  |
| 514 | Who give a decision role in the household? | Mother  Father | | | Others specify-------------- | | |
| 515 | Is there any family member who eats any food item including animal products for the last 24 hour other than the child? | .Yes | 0. No | | | | |
| 516 | Is there any family member who eats at any time without considering fasting time during 24 hours other than the child? | Yes | 0. No | | | | |

Part -6: Household Food Insecurity Access Scale (HFIAS) Measurement Tool

| 601. | In the past four weeks, did you worry that your household would not have Enough food? | Yes 0.No (Skip to Q#603) |
| --- | --- | --- |
| 602. | How often did this happen? | Rarely (once or twice in the past four weeks)  Sometimes (three to ten times in the past four weeks)  Often (more than ten times in the past four weeks) |
| 603. | In the past four weeks, were you or any household member not able to eat the kinds of foods you preferred because of a lack of resources? | **1**.Yes 0.No (Skip to Q#605) |
| 604. | How often did this happen? | Rarely (once or twice in the past four weeks)  Sometimes (three to ten times in the past four weeks)  Often (more than ten times in the past four weeks) |
| 605. | In the past four weeks, did you or any householdmember have to eat a limited variety of foods due to a lack of resources? | 1. Yes 0. No(Skip to Q#607) |
| 606. | How often did this happen? | Rarely (once or twice in the past four weeks)  Sometimes (three to ten times in the past four weeks)  Often (more than ten times in the past four weeks) |
| 607. | In the past four weeks, did you or any household member have to eat some foods that you really did not want to eat because of a lack of resources to obtain other types of food? | Yes 0.No (skip to Q#609) |
| 608. | How often did this happen? | Rarely (once or twice in the past four weeks)  Sometimes (three to ten times in the past four weeks)  Often (more than ten times in the past four weeks) |
| 609. | In the past four weeks, did you or any household member have to eat a smaller meal than you felt  you needed because there  was not enough food? | 1. Yes 0. No (Skip to Q#616) |
| 610 | How often did this happen? | Rarely (once or twice in the past four weeks)  Sometimes (three to ten times in the past four weeks)  Often (more than ten times in the past four weeks) |
| 611. | In the past four weeks, did you or any other household member have to eat fewer meals in a day because there was not enough food? | Yes 0. No (skip to Q#613) |
| 612 | How often did this happen | Rarely (once or twice in the past four weeks)  Sometimes (three to ten times in the past four weeks)  Often (more than ten times in the past four weeks) |
| 613. | In the past four weeks, was there ever no food to eat of any kind in your household because of lack of Resources to get food? | Yes 0. No(skip to Q#615) |
| 614. | How often did this happen? | Rarely (once or twice in the past four weeks)  Sometimes (three to ten times in the past four weeks)  Often (more than ten times in the past four weeks) |
| 615. | In the past four weeks, did you or any household member go to sleep at night hungry because there was not enough food? | 1.Yes 0.No (skip to Q#617) |
| 616. | How often did this happen? | Rarely (once or twice in the past four weeks)  Sometimes (three to ten times in the past four weeks)  Often (more than ten times in the past four weeks) |
| 617. | In the past four weeks, did you or any household member go a whole day and night without eating anything because there was not enough food? | Yes 0.No |

**Part VII: Sanitation information**

| **SN** | **Questions** | **Response** | | | | | |
| --- | --- | --- | --- | --- | --- | --- | --- |
| 701 | Source of water | 1. Tap water 2. Other Specify----------------- | | | | | |
| 702 | water treatment | Yes 0. No | | | | | |
| 703 | If yes for Q702 how did you treat water? | Boiling | | 2.water filter | 3.Wuha Agar | | 4.Other Specify______ |
| 704 | Availability of Toilet | Yes | | No |  | |  |
| 705 | Type of latrine | Private | | Shared | | | 3 Other Specify |
| 706 | Practice of hand washing (more than one response possible) | 1.Afterusing toilet  2.Beforepreparing food | | After preparing food  After cleaning baby defecation | | | 4.Before feeding the baby  5.other specify______ |
| 707 | Materials for hand washing | 1.Soap | 2. Ash | | | 3Only water | |
| 708 | Bath taking of the child? (more than one response possible) | Twice Daily  Daily | Every three day | | | After defecation | |

**Part VIX: The section asks the household assets, economy of the urban**

| No | Questions | Response | | | | | | |
| --- | --- | --- | --- | --- | --- | --- | --- | --- |
| 801 | Do you belong to the house? | 1. Yes 0. No | | | | | | |
| 802 | What is the roof of the main house? | 1.Corrugated iron sheet | | 2.Thatch | | | 3. Other specify --- | |
| 803 | What is the wall of the main house? | 1.Mud | 2.Cement | | 3.Bricks | | 4.Other specify -- | |
| 804. | What is the floor of the main house? | 1.Soil | 2.Cement | | | 3.Ceramic | | 4.Other specify--- |
| 805 | Availability of electricity | 1. Yes 0. No | | | | | | |
| 806 | Availability of radio | 1. Yes 0. No | | | | | | |
| 807 | Availability of television | 1. Yes 0. No | | | | | | |
| 808 | Availability mobile | 1. Yes 0. No | | | | | | |
| 809 | Availability of non-mobile telephone | 1.Yes 0.No | | | | | | |
| 810 | Availability of refrigerator | 1. Yes 0. No | | | | | | |
| 811 | Availability of chair | 1. Yes 0. No | | | | | | |
| 812 | Availability of table | 1. Yes 0. No | | | | | | |
| 813 | Availability of bed with cotton/sponge/spring matters | 1. Yes 0. No | | | | | | |
| 814 | Availability of electric baking stove | 1. Yes 0. No | | | | | | |

THANK YOU FOR YOUR TIME!!!
